# Involvement of IL-26 in bronchiolitis obliterans syndrome but not in acute rejection after lung transplantation

**DOI:** 10.1186/s12931-022-02036-3

**Published:** 2022-05-02

**Authors:** Jesper M. Magnusson, Petrea Ericson, Sara Tengvall, Marit Stockfelt, Bettina Brundin, Anders Lindén, Gerdt C. Riise

**Affiliations:** 1 Department of Respiratory Medicine, Institute of Medicine Sahlgrenska Academy at the University of Gothenburg, Bruna stråket 11, 41345 Gothenburg, Sweden; 2 Division for Lung and Airway Research, Institute of Environmental Medicine, Karolinska Institutet, Stockholm, Sweden; 3 Department of Rheumatology and Inflammation Research, Institute of Medicine, Sahlgrenska Academy at the University of Gothenburg, Gothenburg, Sweden; 4 Department of Respiratory Medicine and Allergy, Karolinska University Hospital, Stockholm, Sweden

**Keywords:** Lung transplantation, Acute rejection, Bronchiolitis obliterans syndrome, Neutrophil, Cytokine, Interleukin-26

## Abstract

**Background:**

The main long-term complication after lung transplantation is bronchiolitis obliterans syndrome (BOS), a deadly condition in which neutrophils may play a critical pathophysiological role. Recent studies show that the cytokine interleukin IL-26 can facilitate neutrophil recruitment in response to pro-inflammatory stimuli in the airways. In this pilot study, we characterized the local involvement of IL-26 during BOS and acute rejection (AR) in human patients.

**Method:**

From a biobank containing bronchoalveolar lavage (BAL) samples from 148 lung transplant recipients (LTR), clinically-matched patient pairs were identified to minimize the influence of clinical confounders. We identified ten pairs (BOS/non-BOS) with BAL samples harvested on three occasions for our longitudinal investigation and 12 pairs of patients with and without AR. The pairs were matched for age, gender, preoperative diagnosis, type of and time after surgery. Extracellular IL-26 protein was quantified in cell-free BAL samples using an enzyme-linked immunosorbent assay. Intracellular IL-26 protein in BAL cells was determined using immunocytochemistry (ICC) and flow cytometry.

**Results:**

The median extracellular concentration of IL-26 protein was markedly increased in BAL samples from patients with BOS (p < 0.0001) but not in samples from patients with AR. Intracellular IL-26 protein was confirmed in alveolar macrophages and lymphocytes (through ICC and flow cytometry) among BAL cells obtained from BOS patients.

**Conclusions:**

Local IL-26 seems to be involved in BOS but not AR, and macrophages as well as lymphocytes constitute cellular sources in this clinical setting. The enhancement of extracellular IL-26 protein in LTRs with BOS warrants further investigation of its potential as a target for diagnosing, monitoring, and treating BOS.

## Introduction

Lung transplantation is an established treatment for end-stage lung disease where no other treatment is available. Short-term patient survival has increased due to improved recipient selection, organ preservation, surgical technique, intensive care management, immunosuppressive treatment and infection control. Long term survival is still limited compared to other solid organ transplants. [1]

Chronic rejection in the form of bronchiolitis obliterans syndrome (BOS) is the main hindrance to long-term survival after lung transplantation [2]. It is a fibro-proliferative process in the small airways leading to airflow limitation and progressive loss of lung function. Notably, chronic rejection was originally defined as pathological obliterative bronchiolitis (OB) [3, 4]. However, the histological confirmation of OB requires bronchoscopy and transbronchial biopsies (TBB), a clinically challenging diagnostic procedure owing to the limited biopsy sizes and the patchy appearance of OB. Therefore, the clinical correlate based on spirometry, BOS, was proposed [5–7]. In essence, BOS is characterized by an obstructive and persistent decline in ventilatory lung function. However, it is apparent that not every case of chronic decline in lung function represents irreversible airway obstruction [8]. Subsequently, the term “chronic lung allograft dysfunction (CLAD)” was introduced to describe any chronic decline, irrespective of its cause. [9]

It is known that repeated acute rejections (AR) during the early postoperative period constitute a substantial risk factor for developing BOS [10, 11]. However, despite extensive research efforts, there is limited understanding of the immunological mechanisms involved in the pathophysiology of BOS and AR. There is evidence that AR involves a specific subset of T helper (Th) cells named Th 17 cells and that these cells respond to donor antigens and subsequently activate antigen-presenting cells such as alveolar macrophages [12]. There is also evidence that BOS development involves interleukin (IL)-17A released from Th17 cells [13]. However, little is known about the role of Th17 cytokines other than IL-17A. This is particularly true for interleukin (IL)-26, a member of the IL-10 family of cytokines, that is produced by Th17 cells, macrophages, and other leukocytes in the airways [14–18]. Importantly, this cytokine contributes to the local mobilization of neutrophils through chemokine release and potentiation of chemotaxis that is induced by other, pro-inflammatory stimuli including the archetype chemokine IL-8 [18].

Given that BOS is associated with local accumulation of IL-8 and neutrophils in the airways and that macrophages are actively involved in AR [19, 20], we hypothesized that BOS and AR both involve IL-26 as well. The pilot study reported in this paper addressed these hypotheses by examining airway samples from patients with AR and BOS using matched control samples for each individual.

## Materials and methods

### Human biobank material

The biobank used in this study consisted of previously collected bronchoalveolar lavage (BAL) samples from diagnostic and protocol bronchoscopies performed during the period 1996–2002 in from 148 lung transplant recipients (LTRs). In the biobank, no other biological samples than BAL was available for analysis. In total, 45% of the patients included in the biobank did develop BOS.

### Samples and matching procedure

All samples with concurrent infection were excluded from the study. Among the remaining, available BAL samples, we matched pairs of patients with respect to pre-transplant diagnosis, age, gender, type of surgery, and sampling time after surgery. We then identified three groups. Firstly, we identified a cross-sectional group of 20 matched pairs consisting of patients, free from acute rejection, with BOS at the time of sampling that were denominated “BOS”, and patients without BOS that did not develop BOS that were denominated “non-BOS”). When multiple samples were available, the first available sample after the BOS diagnose, was selected. The characteristics of these specific LTRs are presented in Table 1. Secondly, we identified 12 matched pairs of patients with biopsy verified AR grade 2 or worse which were denominated “AR”, and patients with no AR which were denominated “non-AR”. No patient in the AR/non-AR analysis developed BOS before sampling and all included samples in the AR group were collected before any specific treatment had been applied. No samples from patients with AR grade 1 were included in any group. All samples from the AR/non-AR matched pairs were collected during the first postoperative year. The characteristics of these LTRs are presented in Table 2. Finally, we identified ten pairs (BOS/non-BOS) with longitudinal LTR samples collected at three subsequent time points, available. One sample was collected as close as possible after the time of BOS diagnosis was established and, for each individual, two samples had been collected before this time point. We used corresponding time intervals for the samples that were collected from the non-BOS group. We used 14 patients from the from the cross-sectional BOS/non-BOS comparison for the longitudinal comparison and six other patients not previously present. The characteristics of this group of LTRs are presented in Table 3. Azithromycin was not introduced as part of the clinical routine at Sahlgrenska University Hospital until 2004. Thus, no BOS patients were treated with Azithromycin at the time of sampling. In total, samples from 70 individuals from the biobank were used. In addition, 13 novel BAL samples were collected and used for flow cytometry and immunohistochemistry (ICC).

**Table 1:** Patient characteristics, matched pairs*, cross-sectional, Non-Bos/BOS

Non-BOS (*n* = 20)				BOS (*n* = 20)				
Diagnosis	Age	Tx type	Gender	Diagnosis	Age	Tx type	Gender	BOS
A1AT	47	SL	M	A1AT	53	SL	F	3b
A1AT	57	SL	F	A1AT	51	BL*	F	2b
CF	31	BL	M	CF	25	BL	M	3b
CF	35	BL	M	CF	21	HL*	M	3b
COPD	47	SL	F	COPD	47	SL	F	2a
COPD	47	SL	F	COPD	53	SL	F	2b
COPD	53	SL	F	COPD	47	SL	F	3a
COPD	53	SL	F	COPD	52	SL	F	2a
COPD	55	SL	F	COPD	55	SL	F	2b
COPD	64	SL	M	COPD	56	SL	M	3a
Eisenmenger	32	HL	M	Eisenmenger	16	HL	M	3b
Eisenmenger	30	HL	M	Eisenmenger	35	HL	M	3a
Eisenmenger	40	HL	F	Eisenmenger	51	HL	F	3b
HUVS	39	BL	F	BAC	47	BL	F	3a
IPF	54	SL	F	IPF	51	SL	F	2a
PAH	22	HL	M	PAH	29	BL	M	3b
PAH	43	BL	F	PAH	26	BL	F	2b
PAH	40	BL	F	PAH	33	BL	F	3a
PAH	42	HL	F	PAH	45	HL	F	3b
PAH	47	BL	M	PAH	51	BL	M	3a

*BOS* bronchiolitis obliterans syndrome, *A1AT* α-1-antitrypsin deficiency, *CF* cystic fibrosis, *COPD* chronic obstructive pulmonary disease, *HUVS* hypocomplementemic urticarial vasculitis syndrome, *BAC* bronchioloalveolar carcinoma, *IPF* idiopathic pulmonary fibrosis, *PAH* pulmonary arterial hypertension, *Tx* transplantation, *SL* single-lung, *BL* bilateral lung, *HL* heart and bilateral lung, *F* female, *M* male

*Matched for pre-transplant diagnosis, age, gender, type of surgery and sampling time after surgery

**Table 2:** Patient characteristics, matched pairs*, cross-sectional, Non-AR/AR

Non-AR (*n* = 12)				AR (*n* = 12)				
Diagnosis	Age	Tx type	Gender	Diagnosis	Age	Tx type	Gender	AR
A1AT	43	SL	F	A1AT	47	SL	F	3
A1AT	48	BL	M	A1AT	47	SL	M	2
A1AT	51	SL	M	A1AT	51	SL	M	2
COPD	53	SL	F	COPD	54	SL	F	2
COPD	53	SL	F	COPD	56	SL	F	2
COPD	57	SL	F	COPD	56	SL	F	2
COPD	58	SL	F	COPD	58	SL	F	2
COPD	62	SL	F	COPD	58	SL	F	2
COPD	62	SL	F	COPD	59	SL	F	2
IPF	22	BL	F	IPF	33	BL	F	2
IPF	49	SL	F	IPF	40	SL	F	2
PAH	40	BL	F	PAH	43	BL	F	2

*AR* acute rejection, *A1AT* α-1-antitrypsin deficiency, *COPD* chronic obstructive pulmonary disease, *IPF* idiopathic pulmonary fibrosis, *PAH* pulmonary arterial hypertension, *Tx* transplantation, *SL* single lung, *BL* bilateral lung, *F* female, *M* male

*Matched for pre-transplant diagnosis, age, gender, type of surgery and sampling time after surgery

**Table 3:** Patient characteristics, matched pairs*, Non-BOS/BOS, Longitudinal

Non-BOS (*n* = 10)					BOS (*n* = 10)				
Diagnosis	Age	Tx type	Gender	Diagnosis		Age	Tx type	Gender	BOS
A1AT	47	SL	F	A1AT		51	BL	F	3b
CF	31	BL	M	CF		25	BL	M	3b
COPD	52	SL	F	A1AT		43	SL	F	2a
COPD	53	SL	F	COPD		47	SL	F	3a
COPD	53	SL	F	COPD		54	SL	F	2a
COPD	58	SL	F	COPD		55	SL	F	2a
COPD	64	SL	M	COPD		56	SL	M	3a
Eisenmenger	31	HL	M	Eisenmenger		51	HL	F	3b
HUVS	39	BL	F	BAC		47	BL	F	3a
PAH	43	BL	F	PAH		29	BL	M	3b

*BOS* bronchiolitis obliterans syndrome, *A1AT* α-1-antitrypsin deficiency; *CF* cystic fibrosis, *COPD* chronic obstructive pulmonary disease, *HUVS* hypocomplementemic urticarial vasculitis syndrome, *BAC* bronchioloalveolar carcinoma, *PAH* pulmonary arterial hypertension, Tx transplantation, *SL* single-lung, *BL* bilateral lung, *HL* heart and bilateral lung, *F* female, *M* male

*Matched for pre-transplant diagnosis, age, gender, type of surgery and sampling time after surgery

### Sampling techniques

All transplanted organs (i.e., lung or heart plus lung) were handled in a similar manner. Surgical procedures and immunosuppression therapies were performed according to existing guidelines at the time of clinical care [21]. The clinical protocol for postoperative follow-up included sampling of transbronchial biopsies and BAL in accordance with the lung transplant program of Sahlgrenska University Hospital [22]. Sampling was also performed when patients displayed clinical signs of worsening, including radiographic infiltrates, fever, dyspnea, hypoxemia, or decline in FEV_1_.

### Bronchoscopy

The collection of bronchoscopy and BAL samples had been performed under local anesthesia in accordance with the clinical protocols of Sahlgrenska University Hospital. The BAL was performed using two infusions with 50 ml of sterile phosphate-buffered saline (PBS) solution (37 °C) into a segmental middle lobe or lingula bronchus, with the bronchoscope in a wedged position. The BAL fluid was re-aspirated after each PBS infusion, collected in a sterile siliconized container, and kept on ice until further processing*.*

### Histological evaluation

The histological evaluation of AR and BOS followed the standard at the time and was verified by an experienced pathologist [23]. According to the established standards at the time, BOS was defined as an irreversible decline in FEV_1_ of at least 20% of the baseline value (i.e., the average maximum FEV_1_ value of two consecutive measurements > 30 days apart during the first postoperative year) [6]. When these samples were collected, the term “chronic lung allograft rejection (CLAD)” and CLAD's different phenotypes had not yet been established. Furthermore, none of the patients was treated with azithromycin. All LTRs in the BOS groups fulfilled the BOS criteria at the time of sampling. Unfortunately, for technical reasons, the material did not allow us to include an analysis of BAL cell differential counts, nor were blood differential counts available or sufficient histological material for additional research.

### Detection of infections

The routine analysis of BAL and TBB specimens for the detection of infectious agents included direct microscopy for cytomegalovirus (CMV) inclusion bodies, Pneumocystis jirovecii, other fungi, and mycobacteria. In addition, immunocytochemistry techniques for pneumocystis, CMV, and Legionella pneumophila were also applied. Conventional cultures for bacteria, including legionella and mycobacteria, fungi, and viruses, were performed, and the presence of CMV and respiratory syncytial virus genome was investigated with polymerase chain reaction (PCR) amplification.

### Cellular sources of IL-26

To identify cellular sources of IL-26 protein, we collected fresh BAL cells from five LTRs diagnosed with BOS and eight LTRs without BOS, who were undergoing routine bronchoscopy at the Sahlgrenska University Hospital.

### Quantification of extracellular IL-26 protein with ELISA

The BAL samples were filtered through a 70um Dacron net (Millipore® Billerica, MA, USA) and centrifuged (378 × *g*, 10 min at 4 °C). The obtained cell-free BAL fluid samples were frozen (− 80 °C) immediately for subsequent cytokine protein analysis with enzyme-linked immunosorbent assay (ELISA).

The protein analysis with commercial ELISA (Cat. No. CSB-E11716h) was performed in accordance with the manufacturer's instructions (Cusabio Biotech®, Co. Ltd, Wuhan, Hubei Province, China). Briefly, diluted samples and reference standards were added to the wells and incubated (2 h). After this, the samples were removed and the biotin-conjugated detection antibody was immediately added and incubated (1 h) without washing. The plates were then washed (3 times) and avidin-conjugated horseradish-peroxidase was added (1 h). Plates were developed with tetramethylbenzidine substrate (30 min), after which a stop solution was added. All the incubations were done at 37 °C. The optical density was quantified (λ 450 nm with 570 nm correction), using a fluorescent microplate reader.

### Identification of intracellular IL-26 protein with flow cytometry

The cells were re-suspended in heat-inactivated human serum in PBS (50 µl, 10%) and incubated (15 min) at room temperature. Without washing, extracellular antibody (2 µl each) was added, including CD3-APC-H7 (Cat. No. 560275, 0.05 mg/ml), CD4-FITC (Cat. No. 555356, 0.5 mg/ml), CD8-FITC (Cat. No. 557085, 0.025 mg/ml), all from BD Biosciences (San Jose, CA, USA), and the cells were then incubated (30 min, 4 °C). After this, the cells were washed with staining buffer. Intracellular staining was performed in accordance with the manufacturer's instructions with the BD transcription factor buffer set (Cat. No. 562574, BD Biosciences). In short, the TF Fix/Perm solution (1 ml) was added, the obtained solution was vortexed and incubated (40 min). Next, the cells were washed with TF Perm/Wash and re-suspended in BD TF Perm/Wash (50 µl) with the intracellular antibody IL-26-APC (Cat. No. IC13751A, 0.01 mg/ml, R&D, Minneapolis, MN, USA) and were then incubated (40 min, 4 °C) in darkness. Finally, the cells were washed with BD Perm/Wash and re-suspended in staining buffer (400 µl). The cells were analyzed in the BD FACSVERSE (BD Biosciences) with FloJo software (FloJo, LLC, Ashland, OR, USA), and 50,000 events were recorded.

### Identification of intracellular IL-26 protein with immunocytochemistry

The ICC staining was performed using cytospin slides prepared with freshly isolated BAL cells harvested from LTRs. These cytospin samples were air-dried and frozen (− 80 °C) until further processing. For study processing, the cytospin slides were thawed and fixed in formaldehyde (4%). To reduce unspecific staining, the slides were incubated first with protein serum-free block (Dako® Agilent Technologies, Denmark), followed by horse serum (5%), and, subsequently, by BLOXALL (Vector Laboratories® Ca, USA). After this, the slides were incubated with primary monoclonal mouse anti-human IL-26 antibodies (5 ug/ml) (Clone 197505, R&D Systems Inc., Mi, USA) or mouse IgG2b isotype control (Clone 20116, R&D Systems Inc.). After washing, slides were incubated with a secondary antibody from the anti-mouse immPRESS kit (5ug/l) (Vector Laboratories®). Bound antibodies were then visualized by ImmPACT VIP-substrate chromogen system (Vector Laboratories®). The procedure was then repeated with in-between washes, but with a primary rabbit anti-human CD68 antibody (5ug/ml) (Abbiotech® Ca, USA), followed by a secondary antibody from the anti-rabbit immPRESS kit (Vector Laboratories®). Bound antibodies were now visualized by ImmPACT DAB-substrate chromogen system (Vector Laboratories®), and slides were finally counterstained with Methyl Green (Vector Laboratories®).

### Statistics

Non-parametric statistical analyses were used throughout the study since the normal distribution of data could not be established with the current sample sizes. The Mann–Whitney *U*-test (GraphPad Prism® software, San Diego, USA) was performed for direct comparisons between groups. Values of *p* < 0.05 were considered to indicate statistical significance.

## Results

In our cross-sectional investigation of BOS/non-BOS (*n* = 20 pairs), we obtained a match for gender in all but one pair, preoperative diagnosis in all but one pair, and type of surgery in all but three pairs (Table 1). The median age difference was 5.8 (0.1–17.4) years and the median time difference for sampling was 3 (0–66) months within the pairs. Neither median age (BOS 46.9 years, non-BOS 45.1 years) nor median sampling time after transplantation (BOS 24 months, non-BOS 24 months) differed markedly.

In our cross-sectional investigation of AR/non-AR (*n* = 12 pairs), we obtained a match for the surgical procedure in all but one pair (Table 2). The median age difference was 3.4 (0.1–10.2) years, and the median time difference for sampling was zero (0–2) months within the pairs. Neither median age (AR 52 years, non-AR 51.5 years) nor median sampling time after transplantation (AR 3.5 months, non-AR 3.5 months) differed markedly between the groups.

As shown in Table 3, in our longitudinal investigation of BOS/non-BOS (*n* = 10 pairs), we obtained a match for preoperative diagnosis in all but two pairs, for the type of surgery in all but one pair, and gender in all but two pairs (Table 3). The median age difference within the pairs was 6.45 (0.9–19.9) years. Here, the median age did not markedly differ between the groups (BOS 49.0 years, non-BOS 50.2 years). The median difference between time points 1 and 2 was seven months in the BOS group and six months in the non-BOS group, and between time points 2 and 3 was 12.5 and 12 months, respectively, for the referred groups.

### Extracellular IL-26 protein

**Fig. 1:**
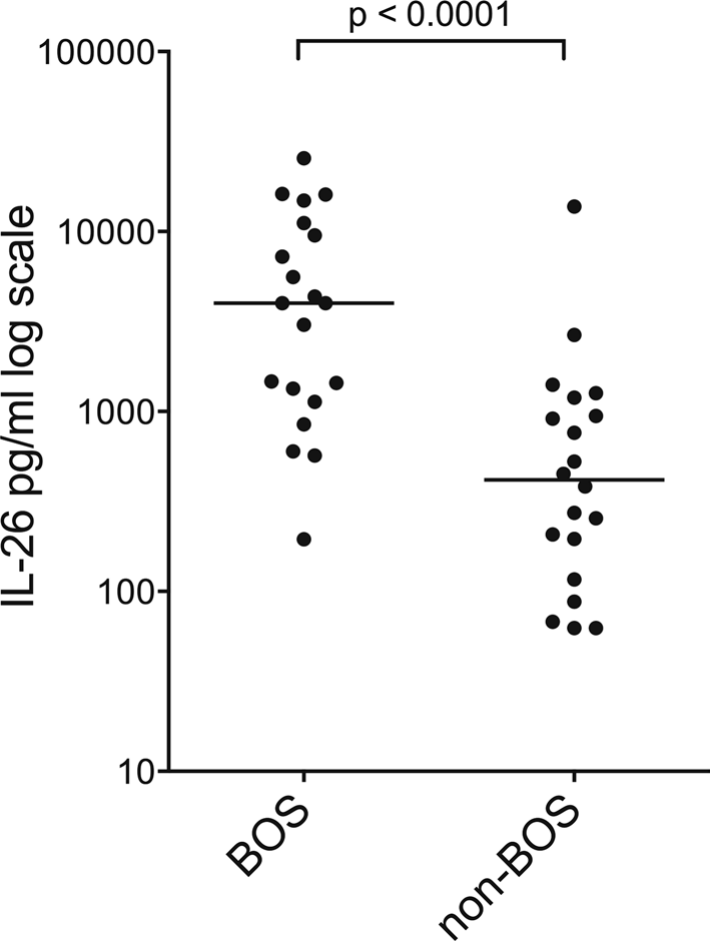
Interleukin (IL)-26 protein concentration (logarithmic scale) in cell-free bronchoalveolar lavage (BAL) fluid from lung transplant recipients (LTRs) with (*n* = 20) and without (*n* = 20) bronchiolitis obliterans syndrome (BOS) quantified by enzyme-linked immunosorbent assay (ELISA). Bars represent the median

In the cross-sectional comparison, the cell-free BAL fluid samples from the BOS group displayed a higher median concentration of IL-26 protein than those in the non-BOS group (*n* = 20 pairs) (shown in Fig. 1).

**Fig. 2:**
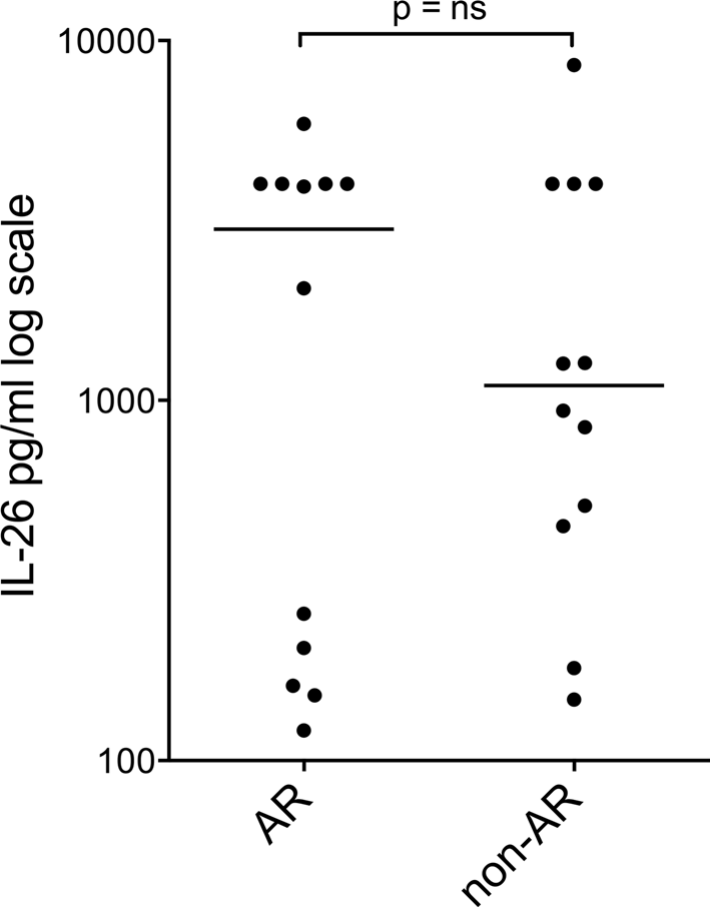
Interleukin (IL)-26 protein concentration (logarithmic scale) in cell-free bronchoalveolar lavage fluid (BAL) from lung transplant recipients (LTRs) with (*n* = 12) and without (*n* = 12) acute rejection (AR) quantified by enzyme-linked immunosorbent assay (ELISA). Bars represent the median

In the cross-sectional comparison, the cell-free BAL fluid samples from patients with or without AR (*n* = 12 pairs) did not show any statistically significant difference (shown in Fig. 2).

**Fig. 3:**
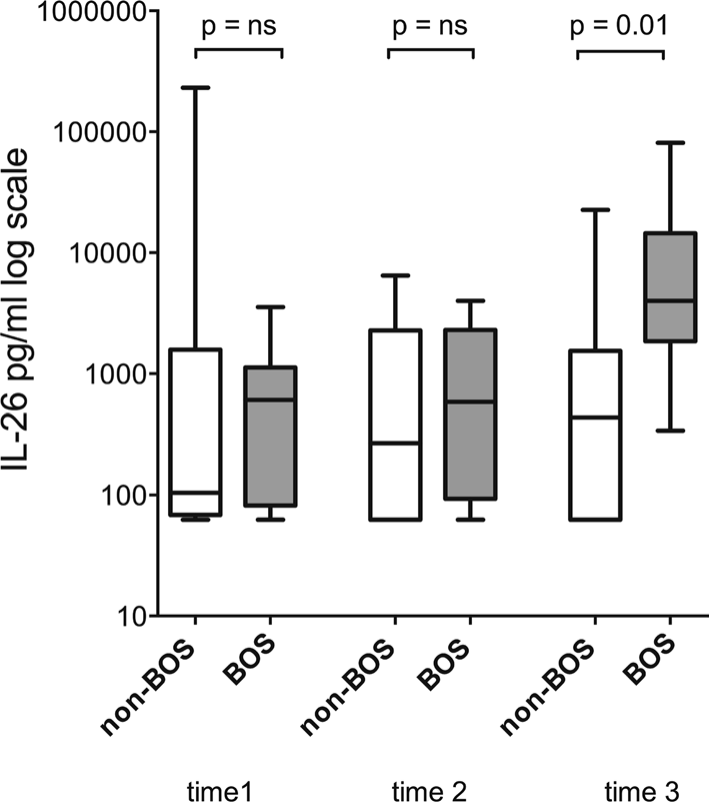
Interleukin (IL)-26 protein concentration (logarithmic scale) in cell-free bronchoalveolar (BAL) fluid from lung transplant recipients (LTRs) with (*n* = 10) or without (*n* = 10) bronchiolitis obliterans syndrome (BOS) quantified by enzyme-linked immunosorbent assay (ELISA). Time one and two before and time three at BOS diagnosis (with corresponding time points in the BOS free group

In the longitudinal comparison, for the cell-free BAL samples from 10 pairs of patients (BOS/non-BOS) with three sampling occasions, the median protein concentration of IL-26 was low in the pre-BOS period in both groups and increased substantially at the time of diagnosis in the BOS group (shown in Fig. 3).

In the cell-free BAL fluid samples collected for analysis of cellular sources of IL-26 (BOS *n* = 5, non-BOS *n* = 6, data not shown), there was a supportive trend towards an increase in median IL-26 protein concentration in the BOS group, but the statistical power was not sufficient to draw a firm conclusion here.

### Intracellular IL-26 protein

**Fig. 4:**
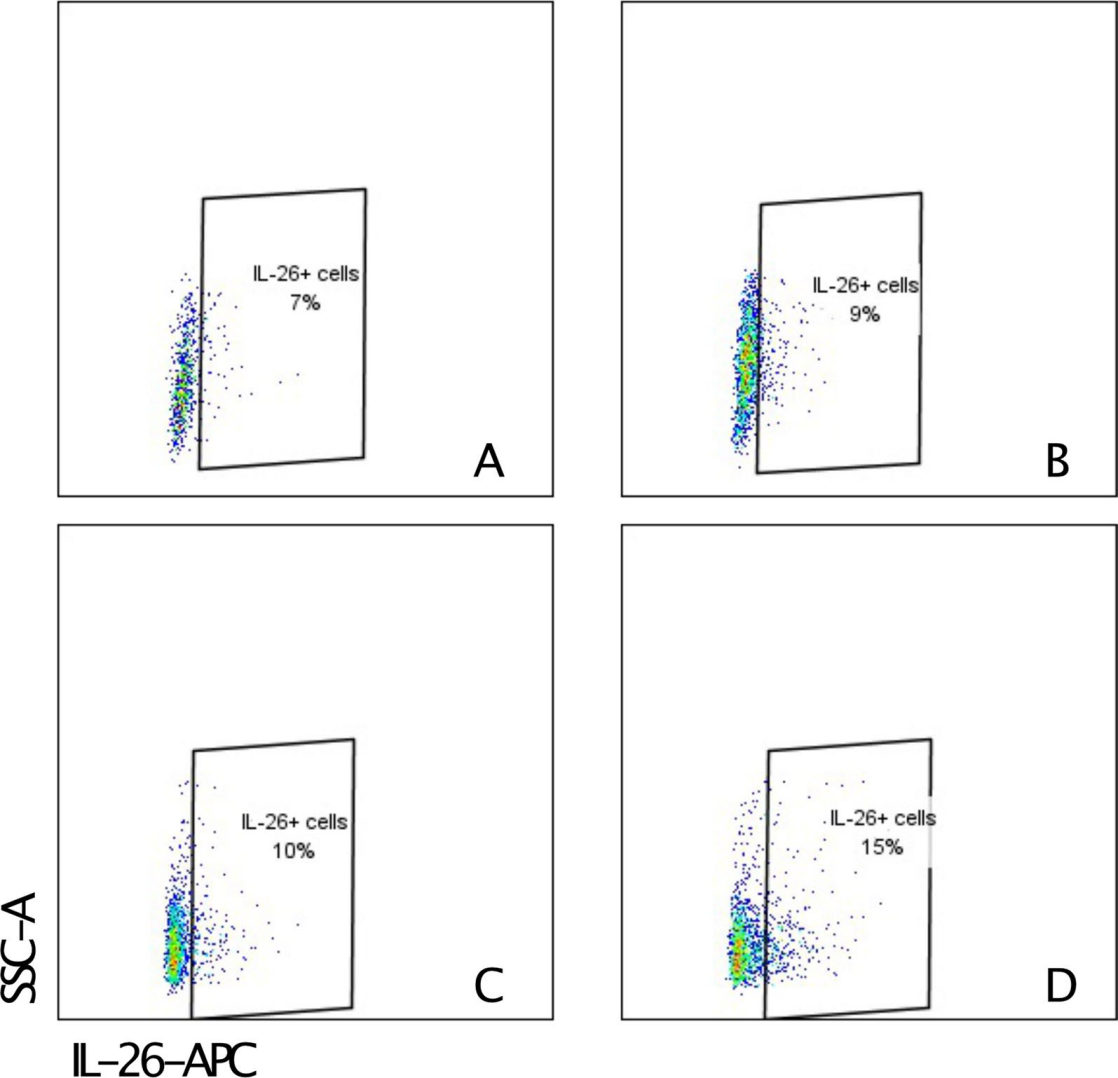
Representative images showing flow cytometry of stained bronchoalveolar lavage (BAL) cells from patients with bronchiolitis obliterans syndrome (BOS), CD4 + cells **A** and CD8 + cells **B** as well as from patients without BOS, CD4 + cells **C** and CD8 + cells (**D**)”

Flow cytometry staining detected IL-26 protein in CD4 + and CD8 + cells from BAL fluid samples in all examined LTR samples. Among CD4 + cells, the median (range) percentage IL-26 positive cells was 6% (4–7) in the BOS group (n = 3) and 4% (4–10) in the non-BOS group (n = 3). Among CD8 + cells, the median (range) percentage IL-26 positive cells was 9% (9–15) in the BOS group and 8% (3–15) in the non-BOS group. Representative flow cytometry images are shown in Fig. 4.

**Fig. 5:**
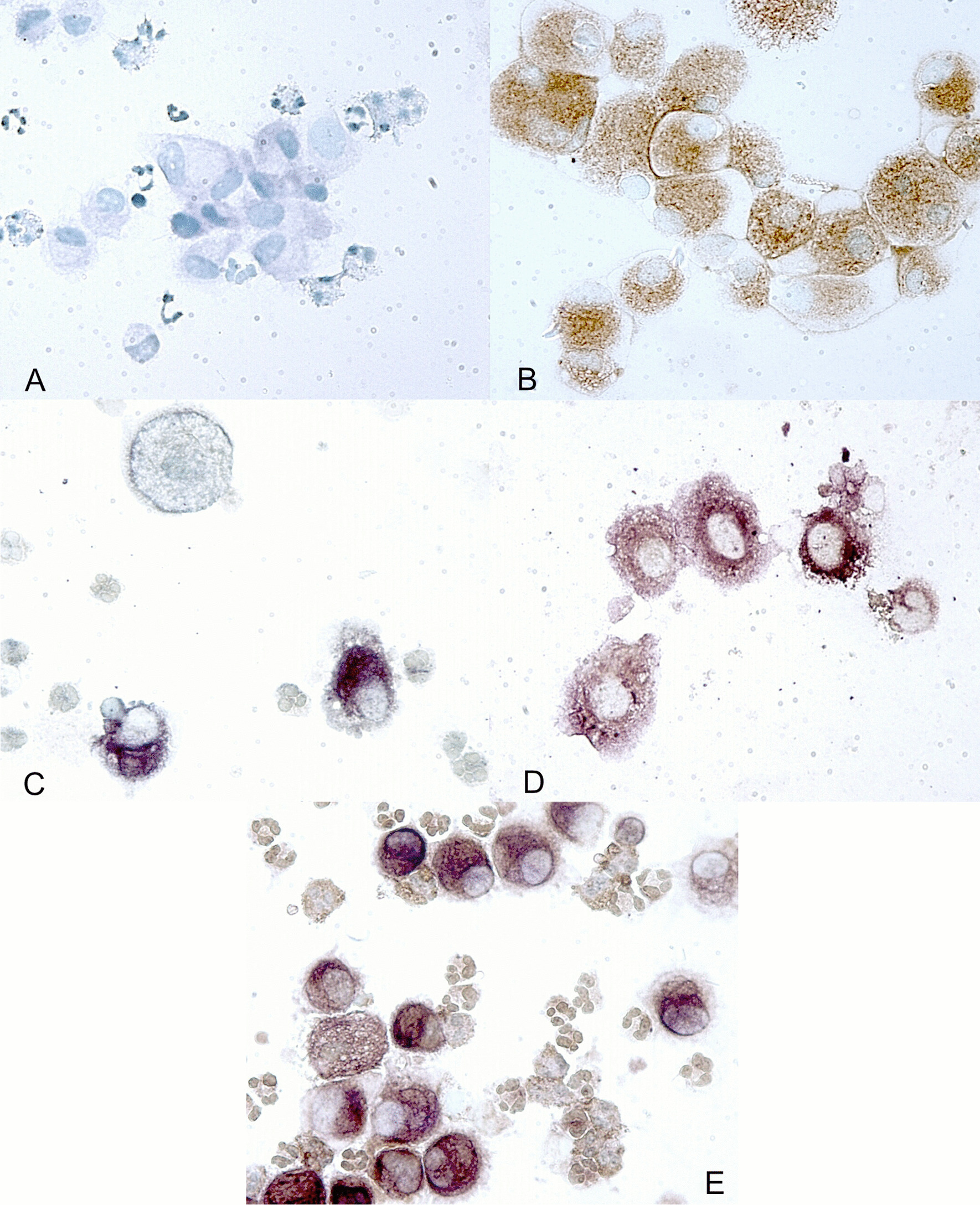
Immunostaining of IL-26 in bronchoalveolar lavage (BAL) cells from lung transplant recipients with (n = 5; **a**–**c** and **e**) and without (n = 4; **d**) bronchiolitis obliterans (BOS). Immunostaining was performed using **a** an IgG2b isotype control antibody, a polyclonal CD68 antibody (b; brown), a monoclonal specific IL-26 antibody (**c**; purple) or a monoclonal specific IL-26 antibody in combination with a polyclonal CD68 antibody (d and e)

Using ICC, we identified strong immunoreactivity for IL-26 protein in predominantly large mononuclear BAL cells from all examined LTRs, in contrast to the isotype control (shown in Fig. 5a, c). In addition, the protocol discriminated between positive and negative cells, arguing for specific binding (shown in Fig. 5c). To ascertain that the IL-26 positive large mononuclear cells were de facto alveolar macrophages, a specific anti-CD68 antibody was added. As expected, this procedure revealed strong CD68 staining in the alveolar macrophages from BAL samples (shown in Fig. 5b). This ICC also confirmed the co-expression signal for CD68 and IL-26 in large mononuclear BAL cells (shown in Fig. 5d, e).

## Discussion

By investigating a well-characterized cohort of LTRs and applying a cross-sectional comparison, we detected higher IL-26 protein concentrations in cell-free BAL fluid samples from BOS patients than in those from non-BOS patients. To examine how the observed difference developed longitudinally, we analyzed BAL samples collected at two time points before and at the time point of BOS diagnosis. We found that the concentrations of IL-26 in both groups were low at both time points in the pre-BOS period and increased in the BOS group at the time of diagnosis. Taken together, these findings indicate that local IL-26 in the airways is enhanced during BOS.

We also collected fresh BAL cell samples to identify cellular sources of IL-26 among airway cells. With flow cytometry, intracellular IL-26 was demonstrated in CD4 + and CD8 + cells from BAL samples of both BOS and non-BOS LTRs. Our investigation with immunocytochemistry demonstrated the presence of IL-26 protein predominantly in large mononuclear cells and small mononuclear BAL cells. Moreover, we demonstrated the co-localization of IL-26 and CD68 in the large mononuclear BAL cells, identifying these cells as alveolar macrophages, whereas the morphology of the small mononuclear cells made it likely that they were lymphocytes.

Given that the LTR samples were collected several years before the current study, we compared the outcome in more recent samples with that in the older samples to evaluate whether the ageing of samples could be a confounding factor of importance for our current study. We found that regardless of the age of the samples there was a higher average concentration of IL-26 protein in the BOS than in the non-BOS group. Thus, these additional results were fully in line with the results from the larger cohort. Given the that we sampled cell-free BAL fluid, stored samples from all study groups during the same period of time, it seems unlikely that our results were caused by ageing of samples.

There is support in the literature for neutrophils playing an important role in the pathophysiology of BOS and for a high neutrophil count being associated with an increase in the archetype chemokine IL-8 [19, 20]. Moreover, IL-26 potentiates neutrophil chemotaxis induced by IL-8 or the bacterial compound *N*-Formylmethionyl-leucyl-phenylalanine in vitro, implying that IL-26 may potentiate neutrophil mobilization towards the source of inflammation and infection [18]. Notably, our current results are well in line with these findings and now add to the growing body of evidence from other studies that IL-26 is involved in chronic inflammatory disorders in human patients, including rheumatoid arthritis (RA) Crohn's disease, asthma, and COPD [14, 24–26]. Moreover, IL-26-producing Th-17 cells may constitute up to thirty per cent of infiltrating T-lymphocytes directly isolated from inflamed lesions of patients with psoriasis vulgaris and RA and in bronchial tissue from patients with severe asthma, a finding that underlines the potential involvement of Th17 cells in the production of IL-26. [27].

In contrast to the case for BOS, we found no substantial and reproducible differences when comparing the concentration of IL-26 protein in cell-free BAL samples from patients with AR to matched controls. Our findings are fully compatible with the fact that AR after lung transplantation in humans is dominated by more of a lymphocytic and less of a neutrophilic inflammation. [28].

It could be argued that the relatively modest sample size in our study constitutes a limitation, although a limited sample size signifies many of the published studies in the logistically complex clinical setting of lung transplantation. However, the sample material used in the current study consisted of carefully matched samples obtained from a much larger sample material. We think that the matching procedure per se effectively minimized the influence of clinical confounders and facilitated the detection of altered cytokine levels related to rejection.

The current study adds conclusive evidence to the previously published studies that support the involvement of IL-26 in chronic inflammatory disorders of the lungs [29, 30], by being the first one to demonstrate the involvement of local IL-26 in a sub-group of LTRs. In this context, it is interesting to note that there is some resemblance between BOS and chronic graft-versus-host disease (cGVHD) from a pathogenic point of view [31]. Furthermore, a recently published study in a murine model of mice transplanted with human umbilical cord blood of cGVHD showed that IL26^+^CD26^+^CD4 T cell infiltration has the potential to play an important role in obliterative bronchiolitis. Moreover, the blockade of caveolin-1 did control pulmonary GVHD by suppressing donor-derived T cells' immune functions, and decreased the production of IL-26 [32]. However, the clinical implementation of data from murine models is uncertain, given the known species differences for the endogenous agonists at the IL-26 receptor complex. [31, 33].

## Conclusion

This study demonstrates that IL-26 is markedly involved and enhanced in BOS but not in AR, implying that IL-26 is involved in the pathophysiology of BOS. To determine whether IL-26 is a useful target for the early detection, monitoring or treatment of BOS, further study with prospective, longitudinal and interventional approaches is needed.

## Data Availability

Data is available upon request.

## Abbreviations

AR: Acute rejection
BOS: Bronchiolitis obliterans syndrome
BAL: Bronchoalveolar lavage
CLAD: Chronic lung allograft dysfunction
cGVHD: Chronic graft versus host disease
ICC: Immunocytochemistry
CMV: Cytomegalovirus
IL: Interleukin
LTR: Lung transplant recipient
PBS: Phosphate-buffered saline
TBB: Transbronchial biopsies
Th: T-helper
